# Pinpointing a Mechanistic Switch Between Ketoreduction and “Ene” Reduction in Short‐Chain Dehydrogenases/Reductases

**DOI:** 10.1002/anie.201603785

**Published:** 2016-07-13

**Authors:** Antonios Lygidakis, Vijaykumar Karuppiah, Robin Hoeven, Aisling Ní Cheallaigh, David Leys, John M. Gardiner, Helen S. Toogood, Nigel S. Scrutton

**Affiliations:** ^1^ Manchester Institute of BiotechnologyUniversity of ManchesterManchesterM1 7DNUK

**Keywords:** biotransformations, isopiperitenone reductase, *Mentha* essential oil biosynthesis, short-chain dehydrogenases/reductases, structure elucidation

## Abstract

Three enzymes of the *Mentha* essential oil biosynthetic pathway are highly homologous, namely the ketoreductases (−)‐menthone:(−)‐menthol reductase and (−)‐menthone:(+)‐neomenthol reductase, and the “ene” reductase isopiperitenone reductase. We identified a rare catalytic residue substitution in the last two, and performed comparative crystal structure analyses and residue‐swapping mutagenesis to investigate whether this determines the reaction outcome. The result was a complete loss of native activity and a switch between ene reduction and ketoreduction. This suggests the importance of a catalytic glutamate vs. tyrosine residue in determining the outcome of the reduction of α,β‐unsaturated alkenes, due to the substrate occupying different binding conformations, and possibly also to the relative acidities of the two residues. This simple switch in mechanism by a single amino acid substitution could potentially generate a large number of de novo ene reductases.

Biosynthetic enzymes involved in the production of menthol oil have been investigated for their biological function and biocatalytic potential because of the high commercial demand for this oil (ca. 31 000 t/US‐$ 373–401 million per year).^1^ Three salutaridine/menthone reductase like subfamilies of short‐chain dehydrogenases/reductases (SDRs)^2^ from *Mentha piperita* were identified, namely (−)‐menthone:(−)‐menthol reductase (MMR), (−)‐menthone:(+)‐neomenthol reductase (MNMR), and isopiperitenone reductase (IPR).1a,1b MMR and MNMR catalyze the ketoreduction of (−)‐menthone **1 a** to (1*R*,2*S*,5*R*)‐menthol **2 a** and (1*S*,2*S*,5*R*)‐neomenthol **2 b**, respectively (Scheme 1).1a Additionally, they act on isomenthone **1 b** to produce (1*R*,2*R*,5*R*)‐neoisomenthol **2 c** and (1*R*,2*S*,5*S*)‐isomenthol **2 d**, respectively. In contrast, IPR catalyzes double bond reduction of isopiperitenone **3 a** to *cis*‐isopulegone **4 a** (Scheme 1).1b

**Scheme 1:**
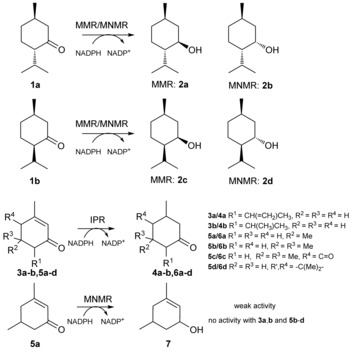
Reactions catalyzed by MMR, MNMR, and IPR.1a,1b

The enzymes of the SDR superfamily are characterized by large sequence divergences (>15 % homology), yet show highly conserved three‐dimensional structures^2^ and an active‐site tetrad typically containing Ser, Tyr, Lys, and Asn.^2^, ^3^, ^4^, ^5^ Interestingly, the three *Mentha* SDRs have high protein‐sequence identities (63–68 %), so we performed comparative studies of MMR, MNMR, and IPR, to pinpoint the determinants of ketoreductase vs. ene‐reductase activity within SDRs.

Kinetic studies of MMR, MNMR, and IPR (see the Supporting Information for details; Figures S1–S3; Table S1)1c and biotransformations were performed with a variety of (a)cyclic, aromatic, and monoterpenoid enones, enals, and enols (Table 1; Figure S4). In some cases, where not commercially available, product standards were synthesized to confirm whether ene reduction or ketoreduction had occurred. Double bond reduction by IPR was seen for six α,β‐unsaturated cyclic enones (**3 a**,**b** and **5 a**–**d**) to produce the corresponding unsaturated ketones (**4 a**,**b** and **6 a**–**d**, respectively; Table 1, entries 1–6; Scheme 1). The highest yields were obtained with the (+/−)‐isopiperitenone mixture **3 a** (50 % *ee*) to produce nearly equivalent amounts of *cis*/*trans*‐isopulegone **4 a** diastereomers. Isophorone **5 b** and ketoisophorone **5 c** were also reduced with high yields and enantiopurity (Table 1, entries 4 and 5). However, the predominant enantiomer of **6 c** generated was (*S*)‐levodione, opposite to (*R*)‐**6 c** generation by the Old Yellow Enzyme (OYEs) ene reductases.^5^, ^6^ Low activity was detected with (*R*)‐piperitenone **3 b** generating enantiopure (*R*)‐pulegone **4 b**. No ketoreduction was observed with any substrate tested.

**Table 1:** Biocatalytic reduction of cyclic ketones by three SDRs.^[a]^

Entry	Enzyme	Substrate	Product	Yield [%]^[b]^	*ee* or *de* [%]^[b]^
1	IPR	**3 a**	**4 a**	91	19 *de* ^[c]^
2	IPR	**3 b**	**4 b**	28	>99(*R*)
3	IPR	**5 a**	**6 a**	44	65 *de* ^[c]^
4	IPR	**5 b**	**6 b**	82	>99^[c]^
5	IPR	**5 c**	**6 c**	77	91(*S*)
6	IPR	**5 d**	**6 d**	16	–
7	MMR	**1 a**	**2 a**	79	90 (1*R*,2*S*,5*R*)
8	MMR	**1 b**	**2 c**	18	83 (1*R*,2*R*,5*R*)
9	MNMR	**5 a**	**7**	5	nd^[d]^
10	MNMR	**1 a**	**2 b**	63	>99 (1*S*,2*S*,5*R*)
11	MNMR	**1 b**	**2 d**	7	72 (1*R*,2*S*,5*S*)

[a] Reactions (1 mL) were performed in buffer (50 mm KH_2_PO_4_ pH 6.0 for IPR; 50 mm Tris pH 7.0 for MMR/MNMR) containing monoterpenoid (**1 a**,**b**, **3 a**,**b**, **5 a**–**d**; 5 mm), enzyme (5 μm), NADP^+^ (10 μm), glucose (15 mm), GDH (10 U), and enzyme (2 μm). The reaction solutions were agitated at 25 °C for 10 h at 130 rpm. Product identification was performed by both comparing retention times with authentic standards and identification by GCMS on a DB‐WAX column (only GCMS identification for product **6 c**). MMR and MNMR data were obtained from previously published work.1c [b] Product yield and enantiomeric excess were determined by GC analysis using DB‐WAX and Chirasil‐DEX‐CB columns, respectively. [c] Lacking enantiopure product standards to assign diastereomeric/enantiomeric identity. [d] nd=not determined due to low product yield.

Biotransformations with MMR and MNMR showed only ketoreduction products, with no detectable double bond reduction (Figure S4).1a,1b Reactions with **1 a** and **1 b** generated the menthol isomers **2 a**–**d** (Table 1, entries 7, 8, 10, and 11). The product *ee* values were medium to high (72 to >99 %). The only other ketoreduction seen was the slow conversion of **5 a** to **7** by MNMR (5 % yield; Table 1, entry 9).

A sequence alignment of the three ketoreductases MMR, MNMR, and salutaridine reductase (SalR; 45–49 % homology to *Mentha* enzymes) from *Papaver somniferum* L with IPR showed each enzyme contained typical SDR‐like motifs, such as those involved in central β‐sheet stabilization, and a TGxxxGhG motif (Figure S5).4b The latter motif in the *Mentha* enzymes contains the motif TGxxKGIG, predictive of a preference for NADP(H) over NAD(H).^7^ A key difference in the sequences between the ketoreductases and IPR was a substitution of the highly conserved catalytic Tyr residue for Glu (238 in IPR). An further sequence alignment of over 500 SDRs revealed only four additional enzymes had substitutions of the active‐site Tyr residue (results not shown). One of these enzymes was IPR from a related *Mentha* sp., which also contained an active‐site Glu. Interestingly, the aldo–keto reductase superfamily contains both ketoreductases (e.g. aldose reductase) and double bond reductases (e.g. Δ^4‐3^‐ketosteroid 5β‐reductase) with high sequence homologies.^8^ In this case, a substitution of an active‐site His for a Glu residue discriminated between ketoreduction and double bond reduction.^9^ Therefore, we investigated the role of the different catalytic acid residues in IPR (Glu 238) and MNMR (Tyr 244) in the reaction mechanism.

We determined crystal structures of both MNMR and IPR (Figure 1), the latter in combination with NADP^+^, alkene **3 a**, and β‐cyclocitral (non‐substrate). Crystallographic methodology, data refinement statistics, and detailed structural descriptions can be found in the Supporting Information (Table S2 and associated discussion). The crystal structures of apo‐IPR and the **3 a**‐ and β‐cyclocitral‐bound complexes were solved by molecular replacement using the known SalR crystal structure (PDB 3O26; 1.2 and 1.7 Å resolution, respectively; Table S2).^10^ The presence of clear density in the *F_0_*−*F_c_* map for NADP^+^ (Figure 1 B) suggested IPR had scavenged it from host cells during protein expression. Substrate **3 a** was bound to the active with the C=C bond close to, and parallel with, the nicotinamide ring of NADP^+^, and close (3.19 Å) to the site of hydride transfer (Figure 1 B right and Figure S 6 a). The carbonyl oxygen atom of **3 a** hydrogen bonds with Glu 238 and the highly conserved Ser 182 and sits at an equal distance (3.15 Å) from both residues. A water molecule hydrogen bonds with Glu 238 and the ribose ring of NADP^+^, suggesting a mechanistic role for this water molecule. Conserved residue Asn 154 is hydrogen‐bonded to Glu 238, while Lys 242 forms hydrogen bonds to the ribose of NADP^+^ and a water molecule, indicating its role in stabilizing NADP^+^ and contributing to a proton relay.^3^ The IPR–β‐cyclocitral co‐crystal structure shows the ligand binds in a non‐active conformation compared to **3 a** binding (Figure S 6 b). No other major changes in residue positions were observed in the co‐crystal structures.

**Figure 1:**
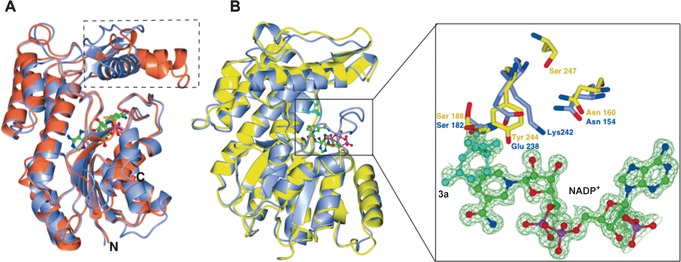
Crystal structure analyses of IPR and MNMR. A) Overlay of IPR (blue; PDB code 5LCX) and SalR (coral; PDB code 3O26) structures. The flap domains of IPR and SalR are indicated by dotted lines. NADP^+^ is displayed as ball and stick and colored by atom type. B) Left: overlay of IPR (gray; PDB code 5LDG) and MNMR (yellow; PDB code 5L53) structures. Right: active site showing side chains of some active‐site residues of IPR and MNMR along with **3 a** (cyan) and NADP^+^. The Figure was prepared using CCP4mg.^11^

The MNMR structure was solved by molecular replacement using IPR as the search model (resolution 2.3–2.7 Å; Table S2), and was found to be structurally similar (rmsd 0.97 Å; Figure 1 B left). A coenzyme‐bound MNMR structure was obtained by soaking crystals with NADP^+^, however no structures were obtained with **1 a**,**b** within the active site. Major structural differences were not observed between apoprotein and NADP^+^‐bound forms. Additional discussion on the crystal structures of the *Mentha* enzymes and related proteins is found in the Supporting Information (Figures S7–S9).

As expected, the conserved Glu 238 of IPR occupied the position of Tyr 244 in MNMR, with a distance between Cβ of **3 a** and the NADPH hydride of 3.18 Å in the co‐crystal structure. Therefore the bulkier MNMR Tyr 244 likely positions substrates in a different conformation compared to that observed for **3 a** in IPR (Figure 1 B right) because of the larger side‐chain bulk of tyrosine. This is consistent with the helix of the flap domain (MNMR) being shifted compared to that in IPR (Figure 1 B right), to accommodate binding of **1 a**,**b**. This structural comparison suggests that this rare residue substitution might be responsible for the switch in activity seen for IPR to NADPH‐dependent 1,4 conjugate reduction of the α,β‐unsaturated carbonyl compound **3 a** to **4 a**.

Based on prior mechanistic studies and our structural studies, we propose mechanisms of action for both ketoreduction (MNMR) and double bond reduction (IPR) in SDRs.^3^, ^4^, ^7^, ^12^ Ketoreduction follows typically an ordered “bi‐bi” mechanism, where the coenzyme binds first and leaves last. MNMR appears to follow this classical SDR ketoreduction mechanism for **1 a** to **2 b** and **1 b** to **2 d** (Scheme 2 A).1a The alcohol product is formed by the transfer of a hydride from NADPH to the carbonyl carbon atom of the substrate with facial selectivity. In the case of SDRs, the 4‐pro‐*S* hydride is transferred, in contrast to MDRs that catalyze 4‐pro‐*R* hydride transfer.4a Concurrent with hydride attack, the carbonyl oxygen atom takes a proton from the conserved Tyr 244 residue acting as a catalytic acid. This starts a cascade of proton transfers through the NADP^+^ coenzyme and Lys 248, terminating with removal of a proton from a water molecule. The conserved Ser 188 residue likely functions to stabilize the substrate, while Lys 248 hydrogen bonds with the nicotinamide ribose moiety, lowering the p*K*_a_ of the Tyr 244‐OH to promote proton transfer.^3^ Residue Asn 160 in SDRs interacts with the conserved Lys 248 and bulk solvent via water molecule(s), forming a protein relay or hydrogen‐bonded solvent network (Scheme 2 A). This likely helps to stabilize the position of Lys 248, thereby assisting the overall ketoreduction mechanism.^3^

**Scheme 2:**
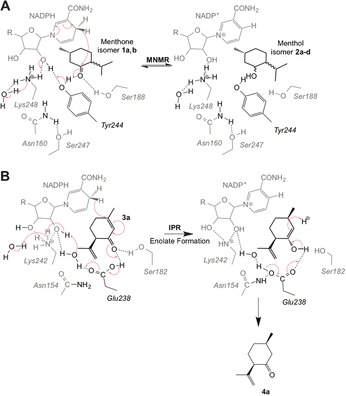
Proposed mechanisms of A) ketoreduction by MNMR and B) reduction of an α,β‐unsaturated double bond by IPR. The three‐dimensional nature of the active sites is represented as compounds in the foreground and background shown in black and grey, respectively.

The structure of the IPR–**3 a** co‐crystal reveals that Glu 238 positions the substrate to allow hydride addition at the C=C bond of **3 a**, rather than the carbonyl carbon atom. In the proposed IPR double bond reduction mechanism, hydride transfer from NADPH to the 4‐position of the α,β‐unsaturated carbonyl system of **3 a** results in formation of the respective enolate ion (Scheme 2 B), which then accepts a proton from the conserved residue Glu 238 to generate the more stable enol. Residue Glu 238 abstracts a proton from a nearby water molecule that may initiate a similar proton transfer cascade to that seen in MNMR. Formation of *cis*‐isopulegone **4 a** then proceeds by Glu 238 abstracting the proton, previously donated to the substrate, resulting in re‐formation of the carbonyl group. Alternatively a nonenzymatic water‐mediated step may occur. Concomitantly, the enolate double bond accepts a proton from water, giving the 1,4 conjugate reduction product (Scheme 2 B). This mechanism is possible in IPR as the side chain of Glu 238, unlike the Tyr side chain, readily dissociates to its conjugate base in water.

To test this hypothesis further, we generated the variants IPR E238Y and MNMR Y244E and performed biotransformation reactions to detect ketoreduction and/or double bond reduction (Table 2). We tested IPR E238Y at pH 6.0, consistent with the preference for lower pH values of the wild‐type enzymes, in addition to reactions at pH 7.0 for comparison with the MNMR Y244E variant. IPR E238Y showed no double bond reduction with any substrate tested (**3 a**,**b** and **5 a**–**d**), however it performed minor ketoreduction with substrate **3 a** to form the equivalent alcohol products **8 a** (Table 2, entries 1 and 2). Additionally it showed MNMR‐like activity towards *Mentha* compounds **1 a**,**b**, forming primarily **2 b** and **2 d**, respectively (Table 2, entries 3–6), although the product yields and enantiopurity were lower than with wild‐type MNMR. Interestingly, reactions with **1 b** at pH 7.0 generated a slightly higher yield of products, but they were obtained in near racemic form (Table 2, entry 6. Therefore, replacing of active‐site Glu by Tyr has converted the enzyme from an ene reductase into a ketoreductase, albeit with lower catalytic efficiency and enantiospecificity.

**Table 2:** Biocatalytic reduction of cyclic ketones by enzyme variants IPR E238Y and MNMR Y244E.^[a]^ 
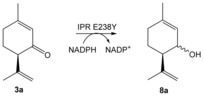

Entry	Enzyme	Substrate	Product	Yield [%]^[b]^	*ee* [%]^[b]^
1 pH 6 2 pH 7	IPR E238Y IPR E238Y	**3 a 3 a**	**8 a 8 a**	<1 <1	nd nd
3 pH 6 4 pH 7	IPR E238Y IPR E238Y	**1 a 1 a**	**2 b 2 b**	38^[c]^ 42^[c]^	45 (1*S*,2*S*,5*R*) 46 (1*S*,2*S*,5*R*)
5 pH 6 6 pH 7	IPR E238Y IPR E238Y	**1 b 1 b**	**2 d 2 d**	33^[d]^ 47^[d]^	47 (1*R*,2*S*,5*S*) *rac*
7 pH 7	MNMR Y244E	**5 c**	**6 c**	3	nd

[a] Reactions (1 mL) were performed in buffer (50 mm KH_2_PO_4_ pH 6.0 for IPR; 50 mm Tris pH 7.0 for MNMR and IPR) containing monoterpenoid (**1 a**,**b**, **3 a**,**b**, and **5 a**–**d**; 5 mm), enzyme (5 μm or 10 μm for IPR and MNMR, respectively), NADP^+^ (10 μm), glucose (15 mm), GDH (10 U), and enzyme (2 μm). The reaction solutions were agitated at 25 °C for 24 h at 130 rpm. Product identification was performed by both comparing retention times with authentic standards and identification by GCMS on a DB‐WAX column (only GCMS identification for product **8 a**). Figure S10 gives the GCMS spectra traces of the additional products and their respective substrates. [b] Product yield and enantiomeric excess were determined by GC analysis using DB‐WAX and Chirasil‐DEX‐CB columns, respectively. nd=not determined due to low product yield. [c] Other isomer formed (20 % yield) was **2 a**. [d] Other isomer formed (2 % yield) was **2 c**.

In the case of MNMR variant Y244E, ketoreduction was not seen with any substrate tested (**1 a**,**b**, **3 a**,**b**, and **5 a**–**d**). Minor double bond reduction was detected with substrate **5 c** to form **6 c** (Table 2, entry 7). MMR and MNMR are known to have narrower substrate specificities than IPR1a (Table 1 and Figure S4), suggesting further mutations are required to form a more active ene reductase.

Interestingly, studies with mechanistically different enzymes of the class I aldolase family (transaldolase and fructose‐6‐phosphate aldolase) have shown that the change of the nature of the catalytic acid/base can have a significant effect on the reaction mechanism.^14^, ^15^ However, the effect of active‐site spacial changes by residue substitution needs to be considered. For example the lack of ketoreduction of wild‐type IPR with **3 a** and **3 b** may be due to a preference for binding in a conformation consistent with double bond reduction, while the steric bulk of Tyr in IPR variant E238Y may orient the substrate in a position suitable for ketoreduction. Further studies will be needed to determine the relative contribution of catalytic residue type vs. steric constraints in determining the overall mechanism of the catalysis.

We have pinpointed a simple mechanistic switch between ene‐reductase and ketoreduction activity in the SDR superfamily. This simple mechanistic switch, in addition to other residue substitutions to improve catalytic efficiency, could potentially transform SDR ketoreductases into novel ene reductases and provide attractive routes to novel ene‐reductase catalysts. This would reduce the dependence on traditional FMN‐containing OYEs for the biocatalytic reduction of α,β‐unsaturated alkenes and complications (reaction rates, yields, and product enantiopurity) that arise when OYEs are affected by molecular oxygen.^13^ Access to a new class of ene reductases would open up the possibility of developing new catalytic specificities typical of the SDR superfamily for the reduction of α,β‐unsaturated alkenes.

## Supporting information

As a service to our authors and readers, this journal provides supporting information supplied by the authors. Such materials are peer reviewed and may be re‐organized for online delivery, but are not copy‐edited or typeset. Technical support issues arising from supporting information (other than missing files) should be addressed to the authors.

Supplementary
